# HPA-1 polymorphism of αIIbβ3 modulates platelet adhesion onto immobilized fibrinogen in an in-vitro flow system

**DOI:** 10.1186/1477-9560-5-2

**Published:** 2007-02-19

**Authors:** Robert Loncar, Volker Stoldt, Sabine Hellmig, Rainer B Zotz, Mario Mihalj, Rüdiger E Scharf

**Affiliations:** 1 Department of Hemostasis and Transfusion Medicine, Heinrich Heine University Medical Center Duesseldorf, Germany; 2 Department of Neurology, University Hospital Firule, Split, Croatia

## Abstract

**Background:**

Platelet adhesion and subsequent thrombus formation on a subendothelial matrix at the site of vascular damage play a crucial role in the arrest of posttraumatic bleeding but also in different pathological thrombotic events, such as acute coronary syndrome and stroke. Recently published studies have clearly demonstrated that platelet integri αIIbβ3 is intimately involved in the occlusive thrombus formation at the site of endothelial damage. Therefore, any genetic variation in the expression of this receptor may lead to an excessive bleeding or excessive thrombus formation. In this study, we evaluated the influence of HPA-1 polymorphism of integrin αIIbβ3 on platelet adhesion onto immobilized fibrinogen using an in vitro system simulating blood flow.

**Methods:**

Platelets in anticoagulated whole blood [49 healthy previously genotyped blood donors) were labelled with fluorescence dye and perfused through a rectangular flow chamber (shear rates of 50 s^-1^, 500 s^-1 ^and 1500 s^-1^). A fluorescence laser-scan microscope was used for visualisation and quantification of platelet adhesion at 15 sec, 1 and 5 minutes after start of perfusion.

**Results:**

During perfusion, the platelet adhesion linearly increased with regard to exposition time and shear rate. Perfusion of blood preincubated with Abciximab over fibrinogen-coated cover-slips showed reduced platelet adherence (absolute fluorescence: 168 ± 35 U vs. 53000 ± 19000 at control experiments, p < 0.05), as well as by perfusion over BSA-coated glass coverslips. Platelet with HPA-1a/1a genotype exhibited initial better adhesion but they also exhibited higher detachment under arterial flow conditions compared to the HPA-1b/1b platelets. Analysis of stable adhesion rate indicate that the platelets carrying the HPA-1b/1b genotype have a higher reactivity threshold for initial interaction with fibrinogen but under the higher shear rate (in regard to time of perfusion) also realize more stable bonds with fibrinogen than platelets with the HPA-1a/1a genotype.

**Conclusion:**

Our data support the contention that genetically determined variants of platelet integrins αIIbβ3 could play a role in arterial thrombogenesis and thus confirm the hypothesis derived from epidemiological studies.

## Background

A platelet-dependent process is the underlying mechanism of arterial thrombosis, and the critical role of platelets in this process is now widely accepted [1,2]. Participation of platelets in arterial thrombosis is centered on the platelet's adhesive properties and the ability to respond to stimuli with rapid activation and, in turn, aggregation [2] – the same features that support the arrest of bleeding from wounds. The normal function of platelets is, however, to arrest bleeding from wounds, which requires adhesion to altered vascular surfaces and rapid cellular activation ensuring the accumulation of circulating platelets and the formation of fibrin in the growing thrombus [1-4].

There are two critical points in thrombus formation: 1) adhesion of inactivated (resting) platelets to subendothelial components such as collagen, deposed vWF or fibrinogen and 2) spreading of activated platelets with consecutive aggregation and thrombus formation. Both processes involve the platelet integrin receptors αIIbβ3. The integrin αIIbβ3 is the most abundant receptor on the platelet membrane surface, at about 80,000 copies per platelet, and is known as the receptor for fibrinogen or von Willebrand factor that mediates platelet adhesion, i.e., the formation of platelet aggregates [5-7]. This receptor is also characterized by several inheritable dimorphisms. The two most common and clinically important β3 alleles encode Leu33 (PlA1 or HPA-1a) and Pro33 (PlA2 or HPA-1b allele of the subunit β3 of the integrin αIIbβ3), with gene frequencies of 0.85 and 0.15, respectively, in the Caucasian population. Clinical studies conducted within the last ten years indicate that there could be a genetically determined predisposition for hyperaggregability which might be mediated by polymorphic receptors including integrin αIIbβ3 involved in platelet adhesion and aggregation [8-14].

Since Weiss et al. [8] for first time in 1996 reported that the gene frequency of the HPA-1b allele of integrin αIIbβ3 was significantly higher among young patients with myocardial infarction compared with age-matched controls, numerous clinical studies were conducted in order to define a role of related polymorphisms in arterial thrombosis. Surprisingly, these studies gave different, and in some cases, contradictory results [8,9,14-16].

Taking into consideration the controversy surrounding these clinical correlations, the goal of this study is to test the functional relevance of HPA-1 platelet receptor polymorphism under standardized microenvironmental conditions using a well-characterized in-vitro flow model.

## Methods and subjects

### Subjects

The study was conducted on 49 healthy blood donors who were previously genotyped. The mean age was 42 ± 12 years, 35 (72%) were men and 14 (28%) were women. None of donors had taken any medication in the preceding 14 days. Screening parameters of hemostasis as well as factors of coagulation were within the normal range according to the international standards. Women on hormone replacement therapy and hormonal contraception, excessive smokers, obese subjects and individuals with positive familiar history related to arterial or venous disease were excluded. Since the frequency distribution of platelet glycoprotein alleles differs with regard to race, only Caucasians were included in the study. This study was performed according to the Helsinki declaration and was approved by the local ethical committee.

### Blood preparation

Venous blood obtained from the cubital vein was immediately anticoagulated with the thrombin inhibitor PPACK (D-phenylalanyl-L-prolyl-L-arginine chloromethyl ketone dihidrochloride, final concentration 40 μM, Calbiochem, San Diego Ca, USA). Platelets were labelled with a fluorescence dye mepacrine (quinacrine dihydrochloride, final concentration10 μM; Sigma Chemical, 60 min at 22°C). It immediately accumulates in the delta granules of platelets but does not influence platelet physiology [17]. Blood was used within two hours of withdrawal. Blood anticoagulated with EDTA and 3.4% sodium citrate was immediately processed for genotyping and for assessment of screening parameters and factors of coagulation, respectively.

### Determination of HPA-1 polymorphism of the β subunit of αIIbβ3

EDTA-anticoagulated blood was immediately centrifuged at 2500 rpm for 10 min at 4°C. Cells were further processed to assess genetic polymorphisms. Genomic DNA was extracted from whole blood with the QIAmp blood kit (Qiagen, Hilden, Germany). After amplification by polymerase chain reaction, genotypes were determined by allele-specific restriction enzyme analysis. Determination of the HPA-1 polymorphism of the β subunit of was performed as previously described [18].

### Preparation of fibrinogen coated cover slips

Glass cover slips (24 × 50 mm) coated with 50 μl of fibrinogen solution (2,5 mg/ml, Sigma-Aldrich) that a sharp interface of the adhesion molecule was formed 10 mm away from the smaller edge of the cover slip [19]. The cover slip was placed in a humid environment (60 min at 37°C) to allow the protein to adhere to the glass surface. Coated cover slips were rinsed with 10 ml of 50 mmol/l phosphate buffered saline (pH 7.35, Serag-Wiessner, Germany) to remove the non-adherent fibrinogen and placed in the flow chamber. Fibrinogen density on the glass surfaces was calculated to be 0.13 μg/mm^2^. For the control experiments cover slips were coated with bovine serum albumin in final concentration of 5 μg/mm^2^. A specificity of binding of platelets to immobilized fibrinogen was tested in experiments (n = 3) with blood preincubated with Abciximab (c7E3, Centocor, Inc; 4 μg/mL, 10 min). c7E3 Fab is a chimerical human/mouse Fab fragment derived from the murine monoclonal 7E3 antibody that binds selectively to the platelet integrin αIIbβ3.

### Flow chamber, perfusion, laser-scan microscopy and data acquisition

Platelets adhesion rates onto fibrinogen and collagen-coated glass coverslips were measured in the rectangular flow chamber under linear shear rate of 50 s^-1^, 500 s^-1 ^and 1500 s^-1^. A shear rate of 50 s^-1 ^represents a venose system, 500 s^-1 ^mimics a wall shear rate of larger arteries and 1500 s^-1 ^represents a typical arteriolar shear rate as well as a shear rate by moderate arterial stenosis [2,4].

A laser-scan microscope (Axiovert 100 M, Carl-Zeiss, Jena, Germany) allowed real-time visualisation of labelled platelets during perfusion through the chamber. To assess time-course of platelet adhesion a series of images (five images per series, 0,7 s pro image) were made at 15 sec, 1 and 5 minutes. Image analysis was performed using the ImageJ software (version 1.26t, NIH, USA). This program allows evaluation of platelet-surface interaction, consecutive aggregation and evaluation of thrombus generation within the defined area of each image. A single frame image corresponded to the area of 980 × 980 μm. The blood was perfused over fibrinogen-coated cover slips as described above. The number of stable, attached platelets on the surface was calculated as number of platelets, which remain their initial adhering position in the first and second image (time frame of 0.7 sec). Platelets were considered to move on the surface when exhibiting a spatial displacement greater than one platelet diameter. To estimate motion, a series of 5 images (time frame 0.7 sec) at one time point were made. Using ImageJ software images were binarised and a threshold was applied to distinguish platelet from background. The first two consecutive frames in a series were superimposed using the logical AND function and the resulting image represented only the overlapping areas of single platelet at two different times.

### Calculations and statistics

Data in text are given as mean values ± SD. The absolute fluorescence was expressed as arbitrary units (pixel units, AU) and represents sum of fluorescence of each thrombus or individual adherent platelet in one defined area. The platelet adhesion was calculated using a logic function of the applied software (ImageJ) and represented a stable platelet adhesion between the first and second image. Taking into consideration that initial platelet adhesion and platelet detachment after initial adhesion is a dynamic process, dependent from shear rate and perfusion time data were normalized. Absolute fluorescence recorded after five minute of perfusion was divided by recorded fluorescence after 1 minute or after 15 sec of perfusion and expressed as a relative adhesion. The relative adhesion represents the increase of absolute fluorescence in function of time and reflects number of stable adherent platelet. Data normalisation allow us to evaluate stability of adherent platelets and to quantify increase of stable platelet adhesion onto immobilized fibrinogen under different shear rates in function of time.

Differences between experimental groups were tested using Student's t-test (two-sided). Regression analyses were based on individual measurements using Spearman's rank correlation coefficient. Statistical analyses were performed using SPSS for Windows, version 6.0.1. *P*-value of less than 0.05 (two-sided) was used to indicate a significant difference.

## Results

Clinical chemistry, haematology and haemostatic laboratory values in the blood obtained from 49 healthy blood donors were within the normal ranges. The mean age was 42 ± 12 years and did not significantly differ between male and female (41 ± 10 vs. 46 ± 12 years, p > 0.05), respectively.

During the perfusion through the rectangular chamber, the platelet adhesion linearly increased with regard to the exposition time at each tested shear rate. In 49 individual experiments, mean platelet adhesion onto fibrinogen-coated coverslips at a shear rate of 50 s^-1^, increased from 15 sec to 5 minutes 3.47 fold (from 3006 ± 1936 AU to 10449 ± 4850 AU), at 500 s^-1 ^9.54-fold (from 4074 ± 1986 AU to 38900 ± 13290 AU) and at 1500 s^-1 ^platelet adhesion increased 14.5 fold (from 3660 AU ± 1917 U to 54500 U ± 22400 AU), respectively.

A specificity of binding of platelets to immobilized fibrinogen was tested bi-directionally with two additional experimental designs. In three experiments a blood was additionally incubated with Abciximab (4 μg/ml for 10 min), c7E3 fragment, which showed a high affinity to the platelet integrin αIIbβ3. A perfusion (1500 s^-1^) of the preincubated blood over fibrinogen-coated cover slips omits to show significant platelet adherence (absolute fluorescence of stable adherent platelet: 168 U ± 35 U vs. 53000 U ± 19000 U at control experiments, p < 0.05). Similarly, in experiments with perfusion of labelled platelets in whole blood over BSA coated glass cover slips no significant adherence was found. The relationship between platelet adhesion and concentration of soluble plasma fibrinogen was not found (p > 0.05). Additional experiments, conducted with supplementation of soluble fibrinogen (up to 9 g/L), omitted to show any difference with regard to the untreated blood (data not present).

To test the influence of the HPA-1 polymorphism of integrin αIIbβ3 on platelet adhesion during perfusion over fibrinogen-coated coverslips blood obtained from 49 blood donors of known HPA-1 genotype (25 with HPA-1a/1a, 12 with HPA-1a1b and 12 with HPA-1b1b genotype) was used in flow experiments.

**Figure 1:**
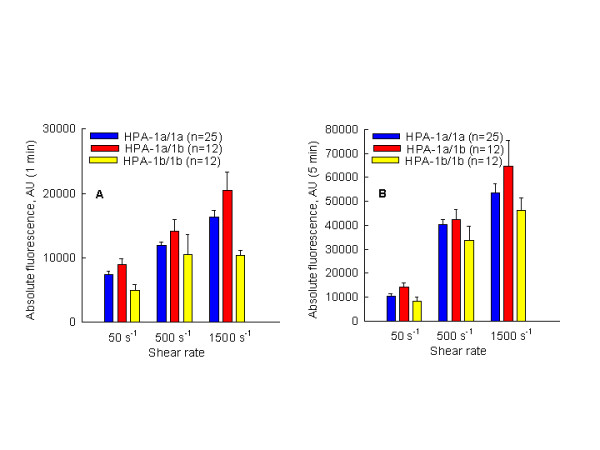
Platelet adhesion onto immobilized fibrinogen under different shear rate conditions with regard to the HPA-1 polymorphism (n = 49). Per each subject 3 flow experiments were conducted (50 s^-1^, 500 s^-1 ^and 1500 s^-1^). At each time point of perfusion (1 min and 5 min) a stack of 5 images was collected and analyzed. Finally 147 perfusion experiments were conducted and 1470 images were analyzed. Each bar represents mean value ± SE.

Platelet adhesion onto immobilized fibrinogen under venous and arterial flow (50 s^-1^–1500 s^-1^) was analyzed after 15 sec, 1 min, and 5 min of perfusion and expressed as absolute fluorescence in arbitrary units (AU). Figures 1A and 1B show platelet adhesion onto immobilized fibrinogen.

Surprisingly, platelets with HPA-1a/1a and HPA-1a/1b genotypes showed higher adhesion activity compared to platelets with the HPA-1b/1b genotype. The significantly higher adhesion activity of platelets with the HPA-1a/1a genotype was observed after 1 min of perfusion at 50 s^-1 ^and at 1500 s^-1^, respectively (p < 0.05, Table 1). A similar trend was also observed at 500 s^-1 ^but without statistical significance.

**Table 1:** Platelet adhesion (mean ± SD) onto immobilized fibrinogen expressed as absolute fluorescence (AU) related to the platelet HPA-1 polymorphism, shear rate and perfusion time.

**Shear rate and perfusion time**	**Platelet adhesion, AU**		
venose shear rate, 50 s^-1^	HPA-1a/1a	HPA-1a/1b	HPA-1b/1b
1 min	7402 ± 2542	8910 ± 2896	4947 ± 2699
5 min	10429 ± 4611	14224 ± 4718	8436 ± 4660
arterial shear rate 500 s^-1^			
1 min	11974 ± 2494	14170 ± 5178	10520 ± 9791
5 min	40319 ± 11255	42424 ± 12344	33581 ± 18756
arterial shear rate 1500 s^-1^			
1 min	16324 ± 5515	20474 ± 8547	10384 ± 2516
5 min	53578 ± 19251	64548 ± 32963	46154 ± 17221

After 5 min of perfusion, a significant difference between platelet adhesion activities with regard to the HPA-1 polymorphism was found only under venous flow (50 s^-1^) between HPA-1a/1b and HPA-1b/1b genotypes (14224 ± 4718 AU vs. 8436 ± 4660 AU, p < 0.05). Even higher platelet adhesion activity of HPA-1a/1a and HPA-1a/1b genotype compared to HPA-1b/1b genotype was also observed under arterial flow, no significant difference was observed neither at 500 s^-1 ^nor at 1500 s^-1^, (p > 0.05, Table 1).

As shown, platelets with HPA-1b/1b genotype after 1 and 5 min of perfusion under arterial flow conditions reached 64% and 86% of the adhesion activity of platelets with the HPA-1a/1a genotype. This dynamic change could indicate that the platelets carrying the HPA-1b/1b genotype have a higher reactivity threshold for initial interaction fibrinogen but also under the higher shear rate (in regard to time of perfusion) also realize more stable bonds with fibrinogen than platelets with the HPA-1a/1a genotype.

To test in which order the HPA-1 polymorphism modulated stability of platelet adhesion onto immobilized fibrinogen the data were "normalized" (see Methods), and a time course of stable platelet adhesion was analyzed with regard to the shear rate and HPA-1 polymorphism.

**Figure 2:**
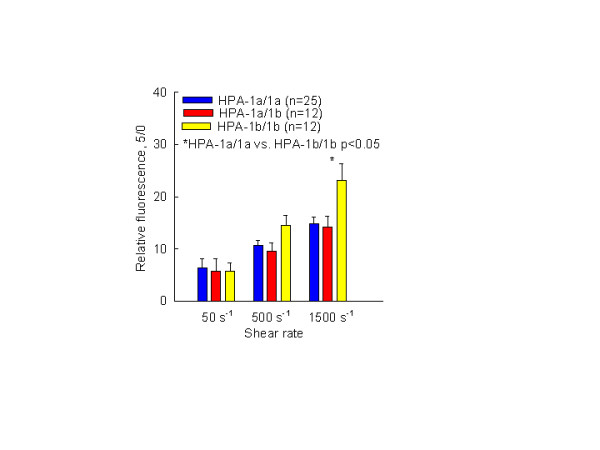
Increase of stable platelet adhesion onto immobilized fibrinogen under different shear rates (50 s^-1^, 500 s^-1 ^and 1500 s^-1^) with regard to the HPA-1 polymorphism within 5 min (each bar represents mean value ± SE). The relative adhesion represents the increase of absolute fluorescence in function of time (absolute fluorescence recorded after five minute of perfusion was divided by recorded fluorescence after 1 minute or after 15 sec of perfusion).

According to the normalized data, an adhesion rate (adhesion between 15 sec and 5 min of perfusion) of platelets with HPA-1b/1b genotype was significantly higher in relation to HPA-1a/1a genotype (Fig. 2). A relative adhesion at shear rate of 1500 s^-1 ^was 23.17 ± 9.9 for 1b/1b genotype versus 14.8 ± 6.5 for 1a/1a genotype, p < 0.05, respectively. A marginal statistical significance was also observed at shear rate of 500 s^-1 ^(HPA-1b/1b 14.45 ± 6.4 vs. 10.66 ± 5.0 for HPA-1a/1a, p = 0.07).

**Figure 3:**
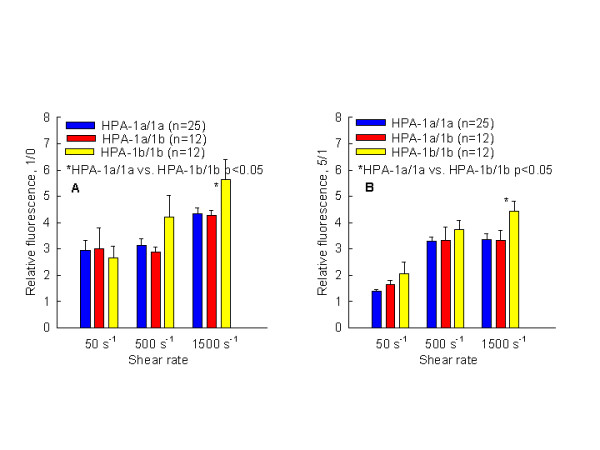
Increase of stable platelet adhesion onto immobilized fibrinogen under different shear rates (50 s^-1^, 500 s^-1 ^and 1500 s^-1^) with regard to the HPA-1 polymorphism within the first 45 seconds of perfusion (Fig. A, "initial phase") and between 1 and 5 min of perfusion ("late platelet adhesion"). Each bar represents mean value ± SE. The relative adhesion represents the increase of absolute fluorescence in function of time (absolute fluorescence recorded after five minute of perfusion was divided by recorded fluorescence after 1 minute or after 15 sec of perfusion).

The time course of platelet adhesion onto immobilized fibrinogen, in regard to the shear rate and genotype was further analyzed. Thrombus growth was analyzed within two different periods of perfusion, at "initial phase" (Fig. 3A) and at "late phase" (Fig. 3B). The time frame between start of perfusion (15 sec, point zero) and one min of perfusion was designated as initial phase and the time frame between min 1 and min 5 as late phase. A statistically significant difference in platelet adhesion with regard to the HPA-1 polymorphism was observed. Surprisingly, the observed difference between different genotype was already present in the initial phase of platelet adhesion (Fig. 3A). The relative adhesion at shear stress of 1500 s^-1 ^was 5.62 ± 2.4 for HPA-1b/1b versus 4.32 ± 1.25 for HPA-1a/1a genotype, p < 0.05, respectively. At 500 s^-1^, a similar trend was observed but without statistical significance (HPA-1b/1b 4.22 ± 2.5 vs. 3.13 ± 1.34 for HPA-1a/1a, p > 0.05). In the late phase (quotient between min 1 and min 5, Fig. 3B), a relative adhesion at shear rate of 1500 s^-1 ^was 4.42 ± 1.21 vs. 3.35 ± 1.17 for 1a/1a genotype, p < 0.05, respectively. A significance was also observed at venous shear rate 2.06 ± 1.32 for HPA-1b/1b vs. 1.39 ± 0.32 for HPA1a/1a, p > 0.05. At 500 s^-1^, a similar trend was observed but without statistical significance (HPA-1b/1b 3.72 ± 1.1 vs. 3.29 ± 0.69 for HPA-1a/1a. Between heterozygous (HPA-1a/1b) and homozygous (HPA-1a/1a) genotype, no significant difference in platelet relative adhesion was observed.

## Discussion

In this study, we now provide experimental evidence that platelet adhesion to immobilized fibrinogen is modulated by platelet receptor polymorphism. This modulation is determined by the specific receptor-ligand interaction and shear rate. In this sense, we provide experimental support for the epidemiological association of the related platelet-receptor polymorphism and arterial thrombosis.

Blood from 49 subjects was collected for use in flow experiments, in order to test the influence of β-subunit polymorphism of integrin αIIbβ3 on platelet adhesion to immobilized fibrinogen. During perfusion, the shear rate expresses "dose"-response and time-response characteristics. The adherent platelets formed a single layer on fibrinogen-coated surfaces. A significant linear increase was observed in platelet adhesion as a function of time, at all shear rates. Image analysis after 1 and 5 minutes of perfusion clearly indicated that the platelets carrying the HPA-1a allele (HPA-1a/1a and HPA-1a/1b genotypes) have higher adhesion activity than platelets carrying the HPA-1b/1b genotype. This difference is continuously detectable at each shear rate applied and at each time point (Figure 1A and 1B) but quantitatively dependent on perfusion time and shear rate. These results are in agreement with the results described by Cadroy et al. [20]. In their study conducted with non-anticoagulated blood, blood was drawn directly from volunteers' cubital veins through a parallel flow chamber. Even though the experimental design differs from ours (shear rates of 650 s^-1 ^and 2600 s^-1^, perfusion time of 2 and 4 min) one common result is evident: platelets with the HPA-1a/1a genotype show higher adhesion activity than platelets with the HPA1b allele. Carlsson et al. also recently reported similar results [21]. The results obtained in a static microtitre-plate system coated with fibrinogen indicated that the adhesion activity of platelets with the HPA-1b/1b genotype is significantly lower compared to the HPA-1a/1a genotype. Interestingly, Cadroy et al. [20], reported that enhanced adhesion activity of platelets with the HPA1a allele observed at 650 s^-1 ^is abolished at shear rates of 2600 s^-1^. In our experiments, the adhesion activity of platelets with the HPA1a allele is constantly higher under venous flow conditions than HPA-1b/1b but varies significantly in regards to perfusion time in experiments at shear rates of 1500 s^-1^. After 1 and 5 min of perfusion under arterial flow conditions, platelets with HPA-1b/1b genotype reached 64% and 86% of the adhesion activity of platelets with the HPA-1a/1a genotype. This observation has initiated an assumption that platelets carrying the HPA-1b/1b genotype have a higher reactivity threshold for initial interaction of αIIbβ3 and fibrinogen but under the higher shear rate (in regard to time of perfusion) also realize more stable bonds with fibrinogen than platelets with the HPA-1a/1a genotype. To test this hypothesis and to omit some interindividual and methodical variation, data were "normalized" (see Methods), and a time course of stable platelet adhesion onto immobilized fibrinogen was analyzed with regard to the shear rate and HPA-1 polymorphism.

As shown in the Result, platelets carrying the HPA-1b/1b genotype demonstrated significantly higher stable adhesion rates between 15 sec and 5 min of perfusion than platelets carrying the HPA-1a/1a genotype (Fig. 2). Stable adhesion at a shear rate of 1500 s^-1 ^was 1.64 times higher compared to the HPA-1a/1a genotype and reached statistical significance. A similar trend was also observed at 500 s^-1^, (14.45 ± 6.4 vs. 10.66 ± 5.0 for HPA-1b/1b vs. HPA-1a/1a, respectively, p = 0.07). At a venous shear rate, this enhanced effect of HPA-1b/1b genotype onto stable platelet adhesion was not observed. An analysis of the platelet adhesion rate of HPA-1b/1b genotype as a function of time (initial and late phase, see Results) showed that the observed difference was present both in the initial phase of the platelet adhesion and in the late phase (Fig. 3A and Fig. 3B).

Based on this observation, it can be concluded that the platelets with the HPA-1b/1b genotype develop stronger adhesive forces than the platelets with the HPA-1a/1a genotype. Statistically significant higher stable adhesion rates of the 1b/1b genotype could be related to the two determinants: 1) steadier platelet/solid surface interactions and 2) reduced displacement of platelets subjected to the shear stress. This results in the formation of thrombi that are more stable than the thrombi formed from HPA-1a/1a. Cadroy also indicated that clot retraction is enhanced by the involvement of platelets with the HPA-1b/1b genotype and that thrombi formed from HPA-1b/1b platelets are more resistant to thrombolysis than the thrombi formed from HPA-1a/1a platelets [22].

An interesting observation was made in light of the behavior of the heterozygous HPA-1a/1b genotype. According to our results, (Fig. 1A and 1B and Fig. 3A and 3B), it appears that platelets carrying "a" and "b" alleles (HPA-1a/1b) have a functional behavior similar to the HPA-1a/1a platelets. Absolute and relative adhesion are comparable with platelets that are homozygous for the "a" allele. Similar to homozygous HPA-1a/1a, our results indicated that platelets carrying the HPA-1a/1b genotype have a lower threshold for adhesion onto immobilized fibrinogen, but they are not capable of producing a stable platelet adhesion as compared to platelets with the HPA-1b/1b genotype. It may be concluded that the functional characteristic of platelets with the HPA-1a/1b genotype is predominantly determined by the "a" allele. This could explain the inconsistency of results between some epidemiological studies in which subjects with different platelet genotype were pooled (for example: HPA-1a/1a vs. HPA-1a/1b plus HPA1b/1b).

In experiments conducted under hemodynamic conditions (shear rate ranging from 25 s^-1 ^to 125 s^-1^) on stabile cell lines (Chinese hamster ovary cells, CHO) with over expression of HPA-1a/1a and HPA1b/1b genotypes, Vijayan et al. [23] also found significant differences in the cell adhesion onto immobilized fibrinogen in regard to the β3 polymorphism. By comparing these results with the results obtained under static conditions [24,25], the authors concluded that a thrombotic potential of respected platelet polymorphism could be underestimated when tested under static conditions. The author provided two indications for a possible explanation for significant differences in adhesion between the two tested cells lines. First, using rhodamine-phalloidin-stained actin, more F-actin was observed at the periphery of the cells with the HPA-1b/1b genotype compared with the HPA-1a/1a genotype, and second, cells with the HPA-1b/1b genotype showed a more robust reorganization of actin. The application of cytohalasin D abolished the differences in cell adhesion in regard to the HPA-1 genotype. Although the experimental design significantly differs compared to our experimental protocol, this report represents one additional experimental proof that HPA-1 polymorphism significantly modulates platelet adhesion and that this modulation is also dependent upon local hemodynamic conditions.

## Conclusion

• Platelet adhesion onto immobilized fibrinogen under arterial flow conditions is mediated by integrin αIIbβ3.

• This specific interaction between immobilized fibrinogen and platelet integrin αIIbβ3 is modulated by the HPA-1 polymorphism of the β-subunit of integrin αIIbβ3.

• The HPA-1b/1b genotype is associated with an increased of stable platelet adhesion.

• This increased adhesion of HPA-1b/1b platelets is already evident within 1 min following the interaction of platelets with immobilized fibrinogen.

Our data support the contention that genetically determined variants of platelet integrins αIIbβ3 could play a role in arterial thrombogenesis and thus confirm the hypothesis derived from epidemiological studies. However, the risk attributable to only a single polymorphism in a complex disease such as stroke or myocardial infarction is probably low. Thus, a single polymorphism may have no clinical significance if evaluated independently, but specific combinations of common polymorphisms and the combination of related polymorphisms might play a relevant role in these diseases. In other words, the thrombotic effect of platelet receptor polymorphisms (single or combined) might be restricted to a combination with microenvironmental risk factors as demonstrated in our study. Understanding of the functional roles of platelet receptor polymorphism may increase our ability to further stratify patients according to their genetically determined thrombosis risk and may give us the opportunity to design and develop individually based therapeutic strategies in the prevention of arterial thrombosis.

## Competing interests

The author(s) declare that they have no competing interests.

## Authors' contributions

RL: designed and coordinated the study and drafted the manuscript.

VS: contributed in the design of the study and performed the flow experiments.

SH: performed the flow experiments.

RZ: participated in the design of the study and in the statistical analysis.

MM: participated in analysis of digital imaging and statistical evaluation

RES: initiated and coordinated the study
